# Understanding the biosynthesis of platelets-derived extracellular vesicles

**DOI:** 10.1002/iid3.66

**Published:** 2015-06-16

**Authors:** Samuel Antwi-Baffour, Jonathan Adjei, Claudia Aryeh, Ransford Kyeremeh, Foster Kyei, Mahmood A Seidu

**Affiliations:** 1 Department of Medical Laboratory Sciences, School of Biomedical and Allied Health Sciences, College of Health Sciences, University of Ghana143, Korle-Bu, Accra, Ghana; 2 College of Agriculture and Natural Sciences, School of Biological Sciences, Department of Molecular Biology and Biotechnology, University of Cape CoastCape Coast, Ghana

**Keywords:** Actin, biomarkers, centrifugation, extracellular vesicles, phosphatidylserine, phospholipids

## Abstract

Platelet-derived extracellular vesicles (PEVs) are described as sub-cellular vesicles released into circulation upon platelets shear stress, activation, injury, or apoptosis. They are considered as universal biomarkers in a wide range of physiological and pathological processes. They are of tremendous significance for the prediction, diagnosis, and observation of the therapeutic success of many diseases. Understanding their biosynthesis and therefore functional properties would contribute to a better understanding of the pathological mechanisms leading to various diseases in which their levels are raised and they are implicated. The review takes a critical look at the historical background of PEVs, their structural components, the mechanism of their formation, physiological, and exogenous stimuli inducing their release and their detection. It concludes by highlighting on the importance of undertaking in-depth studies into PEVs biosynthesis and subsequently gaining a better understanding of their biological role in general.

## Introduction

Extracellular vesicles (EVs), also known as microparticles are a heterogeneous group of small, membrane-coated vesicles with a diameter of 0.1–1 μm 1,2. They are phospholipid rich particles containing certain membrane receptors as well as other proteins inherent in their parental cells 3. Extracellular vesicles are released from the cellular membrane when various types of cells undergo activation or apoptosis and the presence of a cell-specific antigen or combination of antigens allows identification of their cellular origin 4. Platelet derived extracellular vesicles (PEVs) are released from the plasma membrane of platelets and are the most abundant EVs in human blood. Various names have been used to describe these vesicles including microparticles, particles, microvesicles, vesicles, and ectosomes probably because the mechanism involved in their release was not always studied in detail and completely understood 4,5.

The presence of basal levels of PEVs is common in healthy individuals and an increase in their release although a controlled event, is a hallmark of cellular alteration. Therefore, pharmacological modulation of circulating PEV concentrations could become a major therapeutic target in the future 6,7. The field of EVs study has gained interest over the past few years, and is constantly gaining momentum as more people are exposed to the subject 8. Various publications have been released over the years describing the molecular and functional characteristics of EVs particularly PEVs. These suggest the importance of PEVs as a key role player in various cell processes rather than just inert bi-products of cellular activation 7,8.

## Historical Background of PEVs

It has been known since the 1940s that human plasma and serum contained a subcellular factor that facilitates fibrin formation 9,10. Using electron microscopic techniques, Wolf in 1967 demonstrated that activated platelets shed membrane fragments (subcellular factor) and was able to show that this subcellular factor consisted of small vesicles which was originally described as “platelet dust” now “PEVs” 11. These PEVs showed procoagulant activity comparable to that of intact platelets and they were associated with phospholipid-related procoagulant activity known as platelet factor 3 (PF3) 11. The procoagulant activity of these PEVs was therefore designated as platelet factor 3 (PF3) 12. Subsequently, it was shown in vitro that platelet-derived EVs were formed during the attachment of platelets to the vascular wall 13.

In recent years, the interest for PEVs has increased substantially, not only because of their procoagulant properties but also because of the role they are thought to play in inflammation processes and their ability to directly affect endothelial functions 14,15. This ability was demonstrated for the first time in patients with idiopathic thrombocytopaenic purpura (ITP) 16. They are also indicated in a number of autoimmune diseases as well as malaria infection whereby the number of PEVs in the plasma of the affected individual is known to increase 17. Subsequently, PEVs happens to be the most abundant EVs in the blood although EVs in the periphery can also arise from erythrocytes, granulocytes, monocytes, lymphocytes, and endothelial cells, which usually circulate at lower numbers [18,19a]^18^.

### Exosomes

Exosomes were earlier described as vesicles of endosomal origin secreted from reticulocytes. However, in the past few years, several groups have reported the secretion of exosomes by various cell types and have discussed their potential biological functions [19b]. Interest has therefore increased around these vesicles as they appear to participate in several cellular processes such as intercellular communication by serving as vehicles for transfer of membrane and cytosolic proteins, lipids, and RNA between cells, thus affecting normal and pathological conditions. Scientists have, however, recently acknowledged the difficulty of separating exosomes from other types of extracellular vesicles, which precludes a clear attribution of a particular function to the different types of secreted vesicles [20a]. Still, interest in exosomes has intensified after their recent description in antigen-presenting cells and the observation that they can stimulate immune responses in vivo.

## Structural Components of PEVs

All extracellular vesicles are released from the membranes of cells and, therefore, contain cell surface or membrane proteins and some cytoplasmic components (antigenic markers) of their parent cells [20b]. This implies that EVs derived from platelets possess different components as compare to EVs from other cell types 21. This is why markers on the EVs membrane are generally used to identify which cell line they were produced from as different cell lines express different markers and proteins on their surfaces 22,23. The type of stimulus that releases the EVs also determines their constituents; therefore, EVs generated by cell activation processes or apoptosis may contain different cell markers even if they are produced by the same cell type 24.

In general, EVs carry proteins characteristic of their parental cell. PEVs express platelet endothelium adhesion molecule (PECAM-1; CD31), CD62p (P-selectin), glycoproteins IIb–IIIa, P-selectin/CD42a, CD41 (GpIb), and CD63 27. In the same token, EVs shed by polymorphonuclear leukocytes (PMN) express selectins, integrins, complement regulators, human leukocyte antigen 1 (HLA-1), and other markers of neutrophils whereas, monocyte derived EVs express tissue factor and P-selectin glycoprotein ligand-1 (PSGL-1) as well as CD14 (lipopolysacharide receptor) 25. Lymphocytic EVs express CD4 and CD8 with erythrocytes staining for glycophorine A 25,26. Endothelial EVs express CD31, CD34, CD54, CD51, CD146 (S endo 1), CD105 (endoglin), 62E (E-selectin) 28. EVs can also express a different set of surface markers than the precursor cells as has been observed with erythrocyte EVs, but the rules for the incorporation of different proteins into EVs are not completely known (Table1)^27^,^30^.

**Table 1:** A table with different proteins differentially enriched in platelets EVs in comparison to EVs from other cell types

Proteins from platelet EVs	Proteins from EVs from other cell types
Platelet basic protein	14-3-3 protein beta/alpha
Actin, cytoplasmic 1	14-3-3 protein gamma
CD9 antigen	Aminopeptidase N
Fibrinogen beta chain	Catenin alpha-1
Haemoglobin subunit beta	Catenin beta-1
14-3-3 protein zeta/delta	CD151 antigen
Haemoglobin subunit	CD 9 antigen
Gamma-1	CD97 antigen precursor
Integrin alpha-IIb	Claudin-1
Integrin beta-3	Claudin-3
Platelet factor 4	Crumbs homology 2 precursor
Platelet glycoprotein 1b beta chain	Dipeptidyl peptidase 4
Ras-related protein Rap-1b	Galectin -3
Thrombospondin-1	Galectin-4
Fibrinogen alpha chain	GTPase KRas precursor
Fibrinogen gamma chain	Junction plakoglobin
Tropomyosin alpha-4 chain	Lin-7 homolog C
Serum albumin	Macrophage migration inhibitory factor
Myosin light polypeptide 6	Mucin-1 precursor
Multimerin-1	Mucin-13 precursor
Beta-actin-lke protein 2	Mucin-16
Gelsolin	Peroxiredoxin-1
Filamin-A	Phosphatidylethalomine-binding protein 1
Profiling-1	Plexin B2
Platelet glycoprotein 1X	Protaglanding F2 receptor
Erythrocyte band 7 integral membrane protein	Ras-related protein R-Ras precursor
Myosin regulatory light chain 12A	Ras-related protein R-Ras2 precursor
Fermitin family homolog 3	Tetraspanin-1
Talin-1	Transgelin-2

### PEVs are surrounded by a phospholipid bilayer

Platelets are surrounded by a plasma membrane consisting of a phospholipids bilayer, containing phosphatidylserine (PS), phosphatidylethanolamine (PE), phosphatidylcholine (PC), and sphingomyeline (SM) 20,30. In unstimulated or resting platelets, the various phospholipid species are distributed asymmetrically in the bilayer 31. However, this asymmetrical phospholipid distribution is usually disturbed or altered during PEVs formation resulting in the exposure of negatively charged phospholipids such as PS and PE on the PEV surface 19,31,32. The PS surface exposure most likely plays a role in some of the in vivo effects of PEVs since PS efficiently binds coagulation factors and their receptors on cellular surfaces 33.

### PEVs also contain actin

Actin is a globular, ubiquitous, roughly 42kDa protein. It is a monomeric subunit of microfilaments and one of the three major components of the cytoskeleton of a eukaryotic cell present in concentrations of over 100 μM 1,6. There are at least six isoforms of actin in mammals coded for by separate genes and these are further subdivided into three classes (alpha, beta, and gamma) according to their isoelectric point. Alpha actin is found in muscle whereas beta and gamma isoforms are prominent in non-muscle cells 34. Individual subunits of actin are known as globular actin (G-actin), which assembles in F-actin.

Actin participates in many important cellular functions, including muscle contraction, cell motility, production of pseudopodia in phagocytic cells, cell division and cytokinesis, vesicle and organelle movement, cell signalling, the establishment and maintenance of cell junctions, and cell shape 34,35. Actin exists mainly as a fibrous polymer, and is associated with protrusions of the plasma membrane followed by EVs formation 36. It is also cleaved under apoptotic conditions by caspase-3 36.

## Formation of PEVs

Before we look at how PEVs are produced, it is important for us to understand the normal constitution of the cell membrane and how it is maintained in a normal physiologically active cell 7. As referred to earlier, the phospholipid bilayer membrane of cells is composed of highly specific phospholipids that are distributed in a precise manner. A form of asymmetry is constantly maintained in a dynamic manner in that phosphatidylcholine (PC) and sphingomyelin (SM) are located on the external membrane whereas phosphotidylserine (PS) and phosphatidylethanolamine (PE) are positioned on the inner membrane 23.

This asymmetry is actively maintained by various enzymes such as the aminophospholipid-translocase or floppase, scramblase, calpain, and gelsolin 23. These enzymes maintain a dynamic asymmetric steady state and allow membrane phospholipids to move to the outside of the cell membrane while at the same time, the aminophospholipids are redirected to the inner side of the cell membrane 23,37. The conservation of this asymmetry is essential for cell survival and physiology, and is achieved by a combination of transmembrane enzymes that are also implicated in PEV production 35,38.

### The Enzymes involved in PEVs formation

#### Gelsolin

Gelsolin is an enzyme that is specific to platelets only and functions by removing proteins that cap the ends of actin filaments of the platelet cytoskeleton 23. This in turn allows actin to reorganize and hence causes platelet activation and contraction together with PEV generation. The enzyme is activated during increased levels of cytosolic calcium 39.

#### Aminophospholipid translocase

Aminophospholipid translocase is an ATP-dependant enzyme that is involved in the constant transportation of PS and PE from the outer layer of the cell membrane to the inner layer. One molecule of ATP is required for the transfer of each PS molecule. The enzyme is inhibited by increased levels of cytosolic calcium 3.

#### Floppase

Floppase is another ATP-dependent enzyme that is thought to work in conjunction with aminophospholipid translocase. Its main action is to transport lipids from the inner to the outer leaflet of the cell membrane; however, its full function is not currently well understood 23.

#### Lipid scramblase

Lipid scramblase is thought to play an important role in platelet coagulant activity and PEVs production. The function of the enzyme is to allow the movement of phospholipids across the cell membrane. The enzyme is activated during increased levels of intracellular calcium, which simultaneously inhibits Aminophospholipid translocase 30,40. This therefore causes a loss of membrane asymmetry and a stable expression of PS is maintained on the outer membrane, which is a sign of PEVs production 15,23. Patients who have Scott syndrome (defect in lipid scramblase) have shown a defect in platelet coagulation activity as well as a reduction in PEVs. This is due to reduced PS expression on the outer membrane hence reduced production of PEVs proving the importance of the enzyme in PEV formation 23.

#### Calpain

Calpain is an enzyme that belongs to the cysteine proteinase of the papainase family. Calpain is activated during increased levels of cytosolic calcium and when activated is associated with various functions that aid in PEV production such as cleaving of cytoskeletal filaments, facilitating PEV shedding, and activating apoptosis via various pathways 23,31.

### Mechanism of PEV formation

The intracellular mechanisms underlying the stimulus leading to the vesiculation process and actual release of PEVs are as yet not fully understood 4,19. While all subcellular structures may have physiological activities, they likely vary depending upon the array of molecules present 1,5. Experiments have shown that PEV release occurs with both activation and cell death either through apoptosis or necrosis 37,41. PEVs are released from the Platelet cell surface membrane following cellular activation or death by either chemical stimuli such as cytokines, endotoxins, or a physical stimulus such as shear stress 30,42.

On platelet activation, there is a rise in cytosolic calcium [ca^2+^] concentration possibly through the entry of extracellular ca^2+^ via ca^2+^ channels in the plasma membrane which are normally closed at physiologic or resting membrane potential, but are activated (i.e., opened) at depolarized membrane potentials 20,43. This consequently leads to the activation of enzymes such as calpain, gelsolin, scramblase as well as protein kinases. Simultaneously, enzymes such as translocase and phosphatases are inhibited; therefore, resulting in cytoskeletal reorganization, loss of membrane asymmetry, membrane blebbing and hence PEV formation and release 30,42. PEVs have also been shown to be released during apoptosis induced in vitro by growth factor deprivation or by complement deposition 29,38. This membrane modification can cause PS exposure and bleb formation, leading to the extrusion of PEVs which incorporates surface proteins and other contents of platelets 39.

During programmed cell death (apoptosis), the release of PEVs is associated with membrane blebbing, which is a characteristic feature of apoptosis. Blebbing involves a dynamic redistribution of cellular contents, perhaps related to volume stress that occurs as cells die and the activation of Rho associated kinase 1 (ROCK-1) 30,44. ROCK 1 is one of the kinases that have been identified to play a role in membrane blebbing and is thought to be key in the formation of PEVs 30. The ROCK kinases are activated by GTP bound Rho and are important mediators of cytoskeletal modifications such as myosin light chain phosphorylation and actin myosin coupling to the plasma membrane 44. Experiments carried out with mouse fibroblasts and human epithelial breast cancer cells showed a decrease in PEV formation on application of a ROCK 1 activity inhibitor (Y27632) hence suggesting that they indeed play a role in PEV formation in apoptosis 34,35. The caspase inhibitor z-VAD-fmk also blocked the cleavage of ROCK-1 and subsequently membrane blebbing and PEV release thereby suggesting a role of caspases (involved in apoptosis) as well in the activation of ROCK-1 35,45. PEVs release appears to occur late in the death process and may occur concurrently with cell fragmentation and the formation of apoptotic bodies which represent shrunken and collapsed cells with nuclear fragmentation 46.

### Physiological and exogenous stimuli inducing PEV release

Though ectocytosis is a general biological property of most eukaryotic cells, platelets release EVs in response to a variety of stimuli. PEV formation in vitro occurs whenever a stimulus is applied which then induces either platelet activation or apoptosis 19. These stimuli include chemical stimuli, such as cytokines, thrombin, and endotoxin, or physical stimuli, such as shear stress or hypoxia 47. Complement membrane attack complex C5b-9, in the presence or absence of antibodies, Ca^2+^ ionophore A23187, phorbol esters, epinephrine, adenosine diphosphate, and microbial peptides such as formyl-methionyl-leucyl-phenylalanine (fMLP) are potent stimuli for PEV release as well 1,2,48. In addition to many biological stimuli, shear stress is an important mechanical factor inducing EV release in platelets and many cell types. It also should be noted that many stimuli can be additive or even synergistic and not all cells die after EV release particularly if the release is part of normal cellular physiological function 28,46.

### Concept of PS-positive PEV release

The concept of plasma membrane blebbing leading to the shedding of PEVs is based on a transverse migration of anionic phospholipids such as PS from the inner layer to the outer layer of the plasma membrane 32,49. The composition and the distribution of cell membrane phospholipids are highly specific but cellular activation or death processes cause an imbalance in the symmetry of the plasma membrane phospholipid distribution as mentioned earlier which results in the exposure of PS on the outer surface of the PEVs through which they can be identified. Phosphotidylserine has therefore become a dominant marker for identifying PEVs and EVs in general 30.

### Summary of PEVs formation

**Figure 1:**
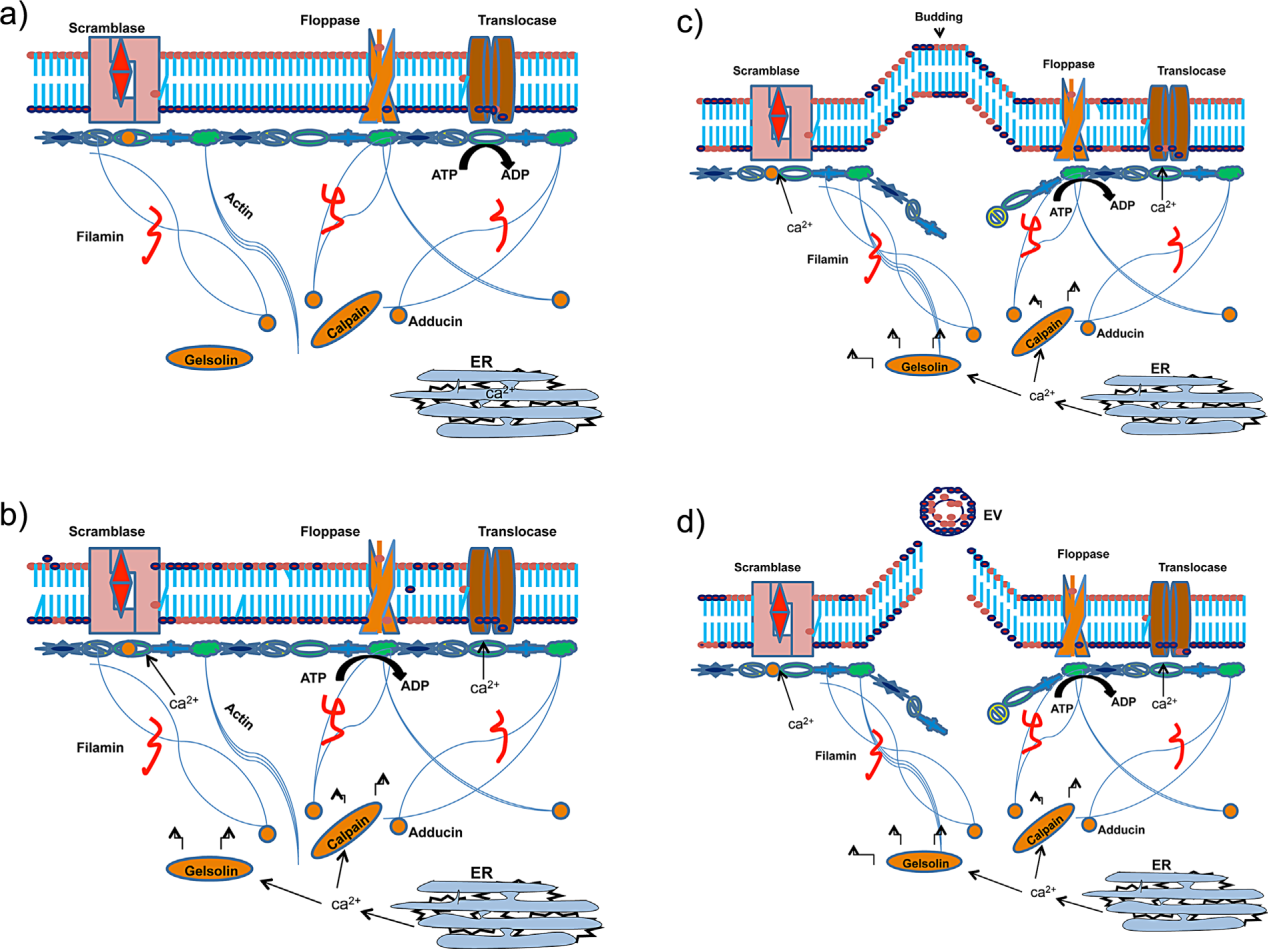
A figure with the different phases depicting how PEVs are formed. (a) *Representation of the resting cell*: Scramblase is inactive while Translocase and Floppase are active maintaining membrane asymmetry. (b) *Cell Activation*: Scramblase, Calpain, and Gelsolin are activated due to calcium release from ER. Calpain cleaves long actin filaments. Gelsolin cleaves actin-capping proteins. Translocase is inactivated. Membrane asymmetry is compromised. (c) *Cytoskeletal Disruption*: protein anchorage to the cytoskeleton is disrupted allowing membrane budding. (d) Extracellular Vesicles (*EVs*) *formation*: EVs are formed and released with increased phosphatidylserine exposed on their external surface.

In resting platelets, scramblase is inactive while translocase and floppase is active, maintaining membrane asymmetry. When platelets are activated, scramblase, calpain, and gelsolin are activated due to calcium release from endothelial reticulum. Calpain then cleaves long actin filaments and gelsolin cleaves actin-capping proteins. Translocase is inactivated and membrane asymmetry is compromised. Protein anchorage to the cytoskeleton is then disrupted, allowing membrane budding which results in PEVs formation and release with PS exposed on their external surface (Fig. 1)^23^.

## Detection of PEVs

Though several methods for analysis of PEVs have been reported, Flow-cytometry and ELISA are the most widely adopted 50. It is, however, difficult to make direct comparison of results because several groups have used different approaches to basic procedures such as centrifugation, re-suspension, and washing of the PEV pellet 23,50. Furthermore, the cell lineage markers adopted are broadly different. Flow cytometry is, however, the method of choice for analyzing and studying of plasma PEVs 50,51. The samples to be tested are obtained from platelet-free plasma after centrifugation although PEVs can be detected in blood samples or fractions thereof as well as in other body fluids such as synovial fluid 6,15.

Using labeled antibodies against platelet-specific antigens and/or activation markers and annexin V, a protein that binds specifically to negatively charged phospholipids in the presence of calcium ions, PEV fractions or subpopulations can be quantified and concurrently their “activation status” established 50. Of each event detected by the flow cytometer, the size (forward scatter-FSC) and density or granularity (side scatter-SSC) are determined electronically, as well as the fluorescence in various channels 2. Fluorescence reflects the amount of antibody bound and, therefore, is an estimate for the amount of antigen exposed on the membrane surface 52. Also, ELISA and electron microscopy technique can be used for PEVs detection and especially for distinguishing PEVs and exosomes. One of the most frequently used ELISAs to quantify PEVs employs a plate coated with annexin V 2,16,52. The detection, quantification, and further analysis of PEVs is very important as the scientific community attempts to understand the biosynthesis of PEVs and, therefore, their biological effects.

## Conclusion

The study of PEVs and EVs in general can still be said to be in the infancy. It is, therefore, important that all aspects of the molecule is studied extensively to aid in the better and complete understating of their biological role in both healthy and disease states. This review has discussed in detail some important aspects of the molecule relating to its biosynthesis in order to emphasize on some latent but important components of the molecule that would assist the scientific community in understanding the physiological and pathological functions of PEVs. Further work on the molecule with particular attention on the constituents discussed, it is believed, will enhance our understating of the biological and molecular functions of it tremendously.
